# Transient hypercapnia reveals an underlying cerebrovascular pathology in a murine model for HIV-1 associated neuroinflammation: role of NO-cGMP signaling and normalization by inhibition of cyclic nucleotide phosphodiesterase-5

**DOI:** 10.1186/1742-2094-9-253

**Published:** 2012-11-20

**Authors:** Jharon Silva, Oksana Polesskaya, Walter Knight, Johnny Ting Zheng, Megan Granger, Tenée Lopez, Fernando Ontiveros, Changyong Feng, Chen Yan, Karl A Kasischke, Stephen Dewhurst

**Affiliations:** 1 Department of Microbiology and Immunology, University of Rochester Medical Center, 601 Elmwood Avenue, Box 672, Rochester, NY 14642, USA; 2 Department of Biostatistics and Computational Biology, University of Rochester Medical Center, 601 Elmwood Avenue, Box 631, Rochester, NY, USA; 3 Department of Medicine, University of Rochester Medical Center, 601 Elmwood Avenue, Box MED, Rochester, NY, USA; 4 Aab Cardiovascular Research Institute, University of Rochester Medical Center, 601 Elmwood Avenue, Box CVRI, Rochester, NY, USA; 5 University of Houston, Houston, TX, USA; 6 Dept. of Neurology, University of Ulm Medical Center, Ulm, Germany

**Keywords:** Cerebrovascular reactivity, HIV-1, Tat-transgenic mice, Hypercapnia, Phosphodiesterase-5

## Abstract

**Background:**

Cerebral blood flow (CBF) is known to be dysregulated in persons with human immunodeficiency virus 1 (HIV-1), for uncertain reasons. This is an important issue because impaired vasoreactivity has been associated with increased risk of ischemic stroke, elevated overall cardiovascular risk and cognitive impairment.

**Methods:**

To test whether dysregulation of CBF might be due to virally-induced neuroinflammation, we used a well-defined animal model (GFAP-driven, doxycycline-inducible HIV-1 Tat transgenic (Tat-tg) mice). We then exposed the mice to a brief hypercapnic stimulus, and assessed cerebrovascular reactivity by measuring 1) changes in cerebral blood flow, using laser Doppler flowmetry and 2) changes in vascular dilation, using in vivo two-photon imaging.

**Results:**

Exposure to brief hypercapnia revealed an underlying cerebrovascular pathology in Tat-tg mice. In control animals, brief hypercapnia induced a brisk increase in cortical flow (20.8% above baseline) and vascular dilation, as measured by laser Doppler flowmetry and in vivo two-photon microscopy. These responses were significantly attenuated in Tat-tg mice (11.6% above baseline), but cortical microvascular morphology and capillary density were unaltered, suggesting that the functional pathology was not secondary to vascular remodeling. To examine the mechanistic basis for the diminished cerebrovascular response to brief hypercapnia, Tat-tg mice were treated with 1) gisadenafil, a phosphodiesterase 5 (PDE5) inhibitor and 2) tetrahydrobiopterin (BH4). Gisadenafil largely restored the normal increase in cortical flow following hypercapnia in Tat-tg mice (17.5% above baseline), whereas BH4 had little effect. Gisadenafil also restored the dilation of small (<25 μm) arterioles following hypercapnia (19.1% versus 20.6% diameter increase in control and Tat-tg plus gisadenafil, respectively), although it failed to restore full dilation of larger (>25 μm) vessels.

**Conclusions:**

Taken together, these data show that HIV-associated neuroinflammation can cause cerebrovascular pathology through effects on cyclic guanosine monophosphate (cGMP) metabolism and possibly on PDE5 metabolism.

## Introduction

HIV-associated neurocognitive disorder (HAND) is characterized by sensory, motor and cognitive dysfunctions that result from HIV infection [1]. HAND remains a major clinical concern despite the widespread use of combination antiretroviral therapy, and a recent study showed that HAND was detected in 52% of HIV-infected persons enrolled in a large multisite patient cohort [2]. HAND represents a continuum of symptoms that may reflect a slowly progressing, multifactorial, degenerative process [3,4]. While these symptoms are thought to be the result of HIV-induced neuroinflammation, the overall pathogenesis of this disease remains incompletely understood.

The effects of HIV-induced neuroinflammation on neuronal structure and function have been extensively studied in both in vitro and in vivo experimental model systems. In contrast, cerebral blood flow (CBF) was initially recognized to be dysregulated in persons with HIV-associated neurologic disease more than 20 years ago [5-10], but remains poorly understood at the mechanistic level. Recently, Ances and colleagues showed that the resting CBF of HIV-infected individuals is significantly decreased in both the lenticular nuclei and the visual cortex [11]. Follow-up studies revealed that HIV infection and aging independently affect functional and resting flow to cortical structures [12], and have shown that resting CBF in persons with HIV infection is reduced to a level equivalent to that of HIV-1 negative persons who are 15 to 20 years older [12]. Not only is resting CBF reduced in the setting of HIV-1 infection [11-13], but cerebrovascular responses to metabolic demand are also perturbed [12]. This may have important implications for neurocognitive function.

To test whether dysregulation of CBF might be due to virally induced neuroinflammation, we used a well-defined animal model in which the pro-inflammatory viral Tat protein is expressed exclusively within the central nervous system (GFAP-driven, doxycycline-inducible HIV-1 Tat transgenic (Tat-tg) mice [14]). To confirm our findings, we also used a second experimental animal model, in which wild-type mice were exposed acutely to HIV-1 Tat by direct intracerebral injection (Tat-ICI; [15]).

We explored the cerebrovascular response of these animals to a defined hypercapnic stimulus, by measuring changes in CBF over the somatosensory cortex using laser Doppler flowmetry, in combination with two-photon in vivo imaging of cortical vessels. We found a significant loss of normal responsiveness to hypercapnia in Tat-exposed mice, relative to controls. This pathology was initially revealed by a long (5 minute) exposure to moderate hypercapnia (6% inspired CO_2_) which was then recapitulated during brief exposure (30 seconds); this transient hypercapnic challenge was selected for subsequent experiments so as to avoid hemodynamic changes as a result of acidosis [16,17], which can occur following longer challenges [18].

In both our chronic (Tat-tg) and acute (Tat-ICI) mouse models for HIV-1 neuroinflammation, the vascular response to brief, moderate hypercapnia was significantly attenuated when compared to control animals (that is, wild-type littermates in the case of Tat-tg mice, or mice injected with saline in the case of Tat-ICI mice). These responses occurred in the absence of changes in cortical microvascular morphology and capillary density in the Tat-tg mice, suggesting that the functional pathology could not be attributed to vascular remodeling.

To explore the mechanistic basis for this pathology, we examined the possible dysregulation of the nitric oxide-cyclic guanosine monophosphate (NO-cGMP) axis within the neurovascular unit, which regulates the hypercapnic dilatory response [19-23]. We next performed experiments to directly address the mechanistic role of the NO-cGMP axis in contributing to the diminished vascular response to brief hypercapnia in mice with HIV-1 associated neuroinflammation. To do this, we exposed Tat-tg mice to hypercapnia in the presence or absence of 1) gisadenafil, a phosphodiesterase 5 (PDE5) inhibitor that prevents degradation of cGMP [24,25], and 2) tetrahydrobiopterin (BH4) which is a limiting cofactor necessary for NO production [26,27]. Treatment with BH4 had little effect on the cerebrovascular response to brief hypercapnia, whereas the PDE5 inhibitor largely restored the normal increase in cortical flow following hypercapnia in Tat-tg mice. Gisadenafil also restored the dilation of small (<25 μm) arterioles following hypercapnia, although it failed to restore full dilation of larger (>25 μm) vessels. This suggests that normalization of flow resulting from (PDE5) inhibition was predominantly determined by the functional recovery of smaller arterioles (<25 μm) within the cortex.

## Materials and methods

### Animal models of HIV-1 induced neuroinflammation

#### Acute model of HIV-1 neuroinflammation

All animal procedures were approved by the University Committee on Animal Research. Adult (10 to 12 weeks old) C57BL/6 male mice were obtained from Charles River Laboratory and housed with a 12-hour light and 12-hour dark photoperiod. Food and water were provided ad libitum. Acute Tat-induced neuroinflammation was produced by stereotactic intracranial injection (ICI) to the right somatosensory cortex, essentially as described [15]. Briefly, a 10-μl microvolume syringe (NanoFil, World Precision Instruments, Sarasota, FL, USA) and 35-gauge needle were silanized (Sigmacote, Sigma, St. Louis, MO, USA) to prevent Tat adhesion. Anesthesia was induced with isofluorane at a rate of 3.5 L/min and maintained at 2.0 L/min in a 50% oxygen and 50% nitrogen gas mixture. A small craniotomy (0.5 mm) was made 1 mm lateral to the sagittal suture at Bregma level −0.5 using a rotary hand tool. Syringe and needle were mounted in an Ultramicropump III syringe pump (World Precision Instruments, Sarasota, FL, USA) that was fixed to a three-axis micromanipulator. The needle tip was then maneuvered into the craniotomy and advanced to a depth of approximately 500–700 μm below cortical surface. Three μl of Tat (recombinant Tat_1-72_, 1 mg/ml, produced and purified from *E. coli*[28]) dissolved in sterile saline (0.9% NaCl), was delivered at a rate of 80 nL/min. This dosage has been shown to elicit a strong neuroinflammatory response in mice following intracerebral injection [15]. As controls, we used 1) sterile saline, 2) Tat inactivated by heating at 95°C for 10 min and 3) 3 μg of recombinant oligomeric HIV-1 Env (HIV-1_YU2_ gp140 [29]). The needle was then removed and the craniotomy was filled with bone wax (Ethicon, Raleigh, NC, USA). Three simple interrupted sutures were used to close the scalp, which was then treated with topical antibiotic. Rimadyl (5 mg/kg) was administered to provide post-surgical analgesia. Forty-eight hours post ICI, cerebrovascular reactivity (CVR) to carbon dioxide was examined.

#### Chronic model of HIV-1 induced neuroinflammation

**Figure 1:**
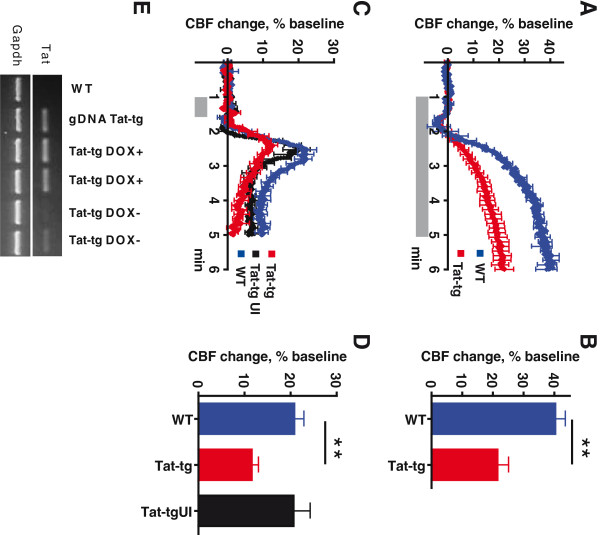
**Tat-transgenic mice display attenuated cerebrovascular response to hypercapnic challenge.** (**A**) Cerebral blood flow (CBF) change in response to 5-minute exposure to 6% CO_2_, as measured by laser Doppler flowmetry (LDF). Results represent mean from seven Tat-transgenic (Tat-tg) mice and four wild type (WT) mice. (**B**) Maximum CBF reached in response to exposure to 6% CO_2,_ for the data shown in (A). **P <0.01, nonparametric permutation test. (**C**) CBF change in response to 30-second exposure to 6% CO_2_, as measured by LDF. Results represent mean from five Tat-tg mice, four Tat-tg mice not induced with doxycycline (DOX) (Tat-tg UI) and five WT mice. (**D**) Maximum CBF reached in response to exposure to 6% CO_2,_ for the data shown in (C). **P <0.01, nonparametric permutation test. (A, C) Values are expressed as a percentage change from baseline CBF, defined here as the mean CBF measured during the one-minute period immediately preceding delivery of CO_2_. Shadowed area along X-axis represents duration of hypercapnic challenge. All data represent mean ± SEM. (**E**) Confirmation of inducible Tat expression in the cortex of Tat-tg mice, as analyzed by RT-PCR. A 141-bp Tat-specific PCR product is shown in the upper panel and a control 480-bp GAPDH product is shown in the lower panel. DNA-free RNA samples were analyzed from a WT c57Bl mouse (negative control), two Tat-tg DOX+ mice, two Tat-tg DOX- mice; genomic DNA from a Tag-tg mouse was used as a positive control (gDNA Tat-tg)

Tat-transgenic (Tat-tg) mice were a generous gift from Drs. Pamela Knapp and Kurt Hauser (Virginia Commonwealth University) [14]. At 8 weeks old, male mice were fed pellets infused with 6 mg/kg doxycycline (Harlan Laboratories, South Easton, MA, USA), ad libitum, for 3 weeks prior to use in experiments. Non-transgenic littermates (WT) were used as controls, and were also fed doxycycline-infused food. The expression of Tat mRNA in cortices of Tat-tg mice exposed to doxycycline was tested by RT-PCR, as described [30]. Figure 1E shows that treatment with doxycycline dramatically increased the level of Tat expression as expected [30,31]. Animals were housed with a 12-hour light and 12-hour dark photoperiod.

#### Drug administration

BH4 (15 mg/kg; Sigma, St. Louis, MO, USA) and gisadenafil besylate (UK-369003, 2 mg/kg; Tocris Bioscience, Bristol, UK) were suspended in a 5% DMSO, 95% physiologic saline solution and was administered by intraperitoneal (IP) injection to mice 2 hours before exposure to hypercapnia; control animals received vehicle alone.

### Cerebral blood flow and physiological parameters

Cerebral blood flow was measured using either bilateral or unilateral laser Doppler flowmetry (LDF) (BLF 21 D, Transonic Systems Inc., Ithaca, NY, USA) over the somatosensory cortex, as described [32]. Physiological parameters were measured as described [32]. Briefly, mean arterial pressure (MAP) was monitored via a femoral artery catheter. Arterial blood gases (ABG) and pH were measured with a blood gas analyzer (Siemens, Rapidlab 248, Erlangen, Bavaria, Germany) in 40 μl microsamples of blood from the femoral artery; blood samples were obtained prior to recording baseline, and after 30 second or 5 minute CO_2_ exposure was complete. The oxygen saturation and heart rate (HR) were continuously monitored using MouseOx (Harvard Apparatus, Starr Life Science Corporation, Holliston, MA, USA). Body temperature was monitored with a rectal probe.

### Cerebrovascular response to hypercapnia

Male mice (25 to 28 g) acutely or chronically exposed to Tat were anesthetized with a urethane/xylazine (1 g/kg, 2 mg/kg, respectively) combination via IP injection. This regimen was chosen to avoid the perturbation of cerebral blood flow that is observed with volatile anesthetics [33]. Animals were placed on a heating pad (37°C) to prevent anesthesia-induced hypothermia, and temperature was monitored rectally for the duration of experiment. Bilateral LDF was used to examine CBF in the acute model of HIV-induced neuroinflammation (in this case, the uninjected left hemisphere served as a control for the injected right hemisphere). Unilateral LDF measurements were recorded for Tat-tg mice. In all cases, initial cortical baseline blood flow was recorded for 1 minute after which 6% CO_2_ was added to a 21% O_2_ air mixture for either 5 minutes or 30 seconds; CBF was then recorded for a total of 9 or 5 minutes, respectively.

### Two-photon microscopy

#### Examination of pial arterioles

Urethane/xylazine anesthetized mice were securely placed into a stereotactic frame and a thin skull window was created as described in [34]. To visualize blood vessels, Texas Red-dextran (MW 70 kDa, Life Technologies, Invitrogen, Grand Island, NY, USA) was injected intravenously (10 mg/kg, in 0.1 ml of saline) into the right femoral vein. Arterioles were identified by the intrinsic auto-fluorescence of smooth muscle (as visualized using a 480/20 bandpass emission filter), branching patterns and blood flow direction. Imaging was performed on a Spectra PhysicsMaiTai HP DeepSee/Olympus Fluoview FV1000 multiphoton imaging setup with a 25× NA 1.05 water immersion microscope objective, and recorded by FluoView1000 software (Olympus America, Center Valley, PA, USA). The excitation wavelength was 880 nm, with the laser power at the sample set below 10 mW. The Texas Red fluorescence was detected using a 607/36 bandpass emission filter. Vessel diameter was extracted from two-photon images of pial arterioles using ImageJ software (NIH). Diameter was measured at two time points - at baseline and again at 30 seconds following exposure to 6% CO_2_.

#### Examination of cortical capillaries

Tat-tg and WT animals were injected with Texas Red-dextran as described above. Dye was allowed to circulate for 5 minutes, and anesthetized animals were then sacrificed by cervical dislocation and decapitated. Intact brains were removed, washed with cold artificial cerebrospinal fluid (aCSF, Harvard Apparatus, Starr Life Science Corporation, Holliston, MA, USA) and placed into a brain slicing matrix (Zivic Instruments, Pittsburgh, PA, USA). Tissue was cut into 2 mm coronal slices and placed onto glass slides with a shallow depression containing aCSF. A glass cover slip was placed over the sectioned tissue and a drop of aCSF was added to the cover slip to provide a liquid interface for the water immersion microscope objective. Images were taken from Bregma level −1.0, interaural 3.10, approximately 1.5 mm from midline. A 25x, 100-μm Z-stack was imaged through the cortex which was then skeletonized and analyzed in three dimensions using AMIRA software (Visage Imaging, San Diego, CA, USA).

#### PDE activity assay

The specificity of the PDE5 inhibitor gisadenafil besylate on PDE5 was evaluated by PDE activity assay on recombinant PDE5A and PDE1A proteins expressed in Cos7 cells as described in [35]. The cGMP PDE activity of Cos7 cell lysates were assayed in buffer containing 20 mM Tris–HCl (pH 7.5), 3 mM MgCl, 15 mM magnesium acetate, 1 μM cGMP, ^3^H]cGMP (100,000 cpm/tube), either 400 mM EGTA (for PDE5A lysates) or 200 mM CaCl and 4 g/ml of CaM (for PDE1A lysates), and indicated concentrations of PDE5 inhibitor gisadenafil. All PDE assay reactions were started by adding the substrate into premixed other components. Reactions were incubated at 30°C for 15 minutes, and then terminated by boiling for 1 minute. After cooling, 2.5 mg/mL snake venom (Sigma, St. Louis, MO, USA) (with 5'-nucleotidase activity) was added to each reaction, and reactions were incubated at 30°C for 10 minutes. Hydrolyzed products were then separated by DEAE-sepharose anionic exchange columns, eluted from columns, and measured via liquid scintillation counter. Enzymatic activity was calculated as percentage total radioactivity minus background, and was established in a linear range prior to initiation of each experiment.

### Statistical analysis

Given the relatively small number of subjects (mice) in each group, it’s difficult to justify the normality assumption of the data distribution. Therefore we used the nonparametric permutation test to compare maximum CBF values reached after exposure to CO_2_ in different groups. To compare arteriole diameters before and after exposure to CO_2_, a two-tailed *t*-test was used.

## Results

### Tat-induced neuroinflammation attenuates the cerebrovascular response to 5 minute and 30 second hypercapnic challenges

The brain vasculature is exquisitely sensitive to changes in tissue and blood levels of carbon dioxide (pCO_2_) [36]. Increased pCO_2_, or hypercapnia, induces a potent global vasodilation in the surface pial arterioles of the neocortex without the need for sensory or motor stimulation [37]. Therefore, we used this dilatory responsiveness as a model to determine whether Tat exposure induces pathology within the vessel itself.

We initially examined responses in Tat-tg mice. Cerebral blood flow was measured by laser Doppler flowmetry, with unilateral placement of the laser Doppler probe (since both hemispheres expressed Tat). Baseline was established for 1 minute after which animals were exposed to 6% CO_2_ for 5 minutes (Figure 1A, the shaded area on the time line shows the period during which animals were exposed to CO_2_). The peak vasodilatory response to hypercapnia was significantly attenuated (*P* = 0.01; nonparametric permutation test) in Tat-tg (21.5% increase in CBF) compared to WT mice (40.3% increase in CBF) (Figure 1B).

Since long hypercapnic challenges have the potential to cause acidosis [18] and resulting hemodynamic changes [16,17], we measured physiologic parameters before and after the 5-minute exposure to 6% CO_2_ (Table 1). There were no differences between Tat-tg, non-tg littermates (WT) or Tat-tg mice not induced with DOX (Tat-tg UI). However, after a 5 minute exposure to 6% CO_2,_ mice developed moderate acidosis. We therefore next assessed whether a more transient exposure to CO_2_ would produce a similar CBF response without causing significant physiological changes. Table 1 shows that short (30 seconds) exposure minimized the magnitude of acidosis, while still eliciting a robust increase in cortical flow (Figure 1C). Comparison of the peak response of Tat-tg (11.6% increase in CBF, above baseline) and WT (20.8% increase in CBF, above baseline) mice to a brief hypercapnic challenge (Figure 1D) revealed a statistically significant difference (*P* = 0.01; nonparametric permutation test). The peak response of Tat-tg UI mice to a brief hypercapnic challenge (20.7% increase in CBF, above baseline) was not different from response of WT mice.

**Table 1:** **Physiological parameters in mice before and after exposure to 6% CO**_**2**_

		**MAP, mm Hg**		**Arterial blood pH**		**PaCO**_**2**_**, mm Hg**	**PaO**_**2**_**, mm Hg**
**Group (N)**	**Exposure to CO**_**2**_	**Before**	**After**	**Before**	**After**	**Before**	**Before**
**WT (4)**	5 min	74.72 ± 4.48	75.52 ± 3.85	7.36 ± 0.02	7.19 ± 0.04	36.33 ± 1.25	96.5 ± 2.65
**Tat-tg (7)**	5 min	72.86 ± 3.45	71.94 ± 3.66	7.34 ± 0.01	7.19 ± 0.03	37.68 ± 1.79	100.88 ± 2.65
**WT (15)**	30 sec	73.29 ± 1.67	71.19 ± 1.65	7.35 ± 0.01	7.27 ± 0.02	35.21 ± 0.65	103.7 ± 1.84
**Tat-tg (16)**	30 sec	75.63 ± 1.74	76.09 ± 1.79	7.35 ± 0.01	7.28 ± 0.01	35.69 ± 1.19	102.98 ± 2.65
**Tat+BH4 (3)**	30 sec	73.2 ± 2.93	73.0 ± 2.48	7.34 ± 0.02	7.28 ± 0.01	36.9 ± 2.25	103.36 ± 10.7
**Tat+PDEi (12)**	30 sec	75.74 ± 2.39	74.72 ± 2.01	7.36 ± 0.03	7.25 ± 0.03	35.52 ± 0.83	104.1 ± 1.93
**Tat+Combo (3)**	30 sec	75.03 ± 5.87	73.43 ± 4.72	7.35 ± 0.02	7.26 ± 0.02	37.77 ± 1.27	108.7 ± 5.27
**WT+Combo (3)**	30 sec	74.2 ± 1.01	71.27 ± 0.74	7.34 ± 0.01	7.21 ± 0.04	38.57 ± 2.65	104.57 ± 10.19
**Tat-tg+Vehicle (4)**	30 sec	73.3 ± 0.87	74.58 ± 1.04	7.36 ± 0.02	7.28 ± 0.01	34.90 ± 1.7	101.45 ± 1.96
**Tat-tg UI (6)**	30 sec	81.35 ± 2.69	78.4 ± 2.34	7.35 ± 0.03	7.27 ± 0.02	40.55 ± 4.54	99.73 ± 1.94
**Tat-injected (4)**	30 sec	79.95 ± 4.66	78.23 ± 4.70	7.38 ± 0.02	7.25 ± 0.03	37.15 ± 1.67	101.25 ± 1.40
**gp140-injected (4)**	30 sec	74.33 ± 4.70	72.78 ± 4.42	7.36 ± 0.01	7.26 ± 0.01	36.03 ± 1.56	106.05 ± 5.42

PDEi, gisadenafil; Combo, combined PDEi and BH4; MAP, mean arterial pressure; PaCO_2,_ partial arterial pressure of CO_2_;

PaO_2,_ partial arterial pressure of O_2_; UI, Uninduced. Data shown as mean ± SEM.

### Tat-induced neuroinflammation attenuates the cerebrovascular response in intracranially injected c57BL/6 mice

**Figure 2:**
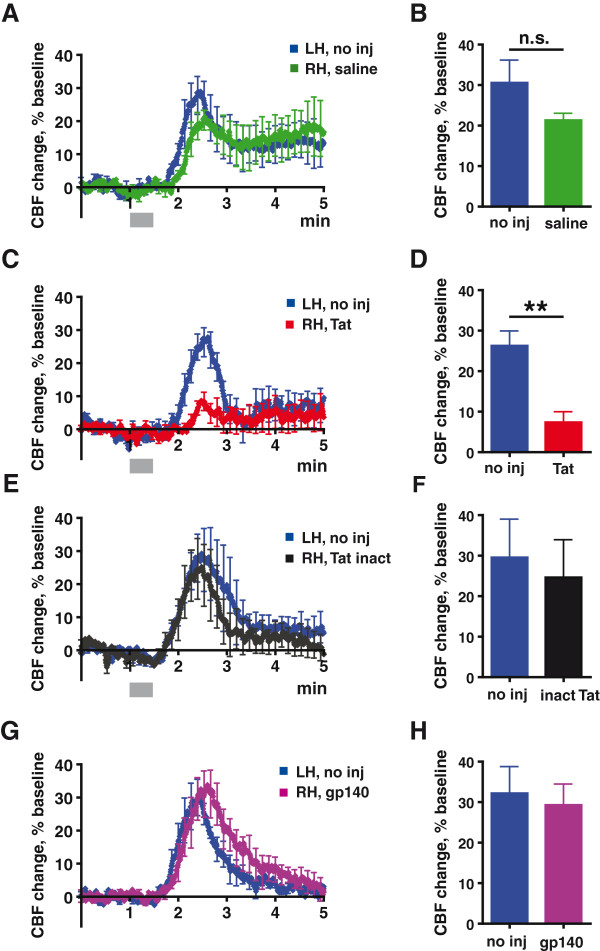
**Acute exposure to Tat dysregulates cerebrovascular response to hypercapnic challenge.** In all panels, cerebral blood flow (CBF) change in response to 30-second exposure to 6% CO_2_, was measured by bilateral laser Doppler flowmetry (BLDF), in c57Bl mice. (**A**) CBF change 48 hours after injection with saline into right hemisphere (RH). Intact left hemisphere (LH) serves as a control. Results represent mean values from three mice. (**B**) Maximum CBF reached in response to 6% CO_2,_ for the data in (A). n.s.: not significant; nonparametric permutation test. (**C**) CBF change 48 hours after injection with Tat into RH. Intact LH serves as a control. Results represent mean values from four mice. (**D**) Maximum CBF reached in response to 6% CO_2,_ for the data in (C). **P <0.01, nonparametric permutation test. (**E**) CBF change 48 hours after injection with heat-inactivated Tat into RH. Intact LH serves as a control. Results represent mean values from four mice. (**F**) Maximum CBF reached in response to 6% CO_2,_ for the data in (E). (**G**) CBF change in mice 48 hours after injection with recombinant HIV-1 Env into RH. Intact LH serves as a control. Results represent mean values from four mice. (**H**) Maximum CBF reached in response to 6% CO_2,_ for the data in (G). (A, C, E, F) Values are expressed as a percentage change from baseline CBF, defined as the mean CBF measured during the one- minute period immediately preceding delivery of CO_2_. The shadowed area along the X-axis represents the duration of the hypercapnic challenge. All data represent mean ± SEM

To determine whether the vascular response to hypercapnia was altered in an acute model of HIV-induced neuroinflammation [15], c57BL/6 mice age 8to 12 weeks, were anesthetized and administered one of the following by stereotactic intracerebral injection to the cortex of the right hemisphere (RH): 1) 3 μl of saline (0.9% NaCl), 2) 3 μl (1 mg/ml) of recombinant HIV-1 Tat in saline, 3) the same amount of heat-inactivated Tat in saline, or 4) 3 μl (1 mg/ml) of recombinant oligomeric HIV-1 Env in saline (HIV-1_YU2_ gp140). The cortex of the left hemisphere (LH) was not injected. Two days later, the response to hypercapnia was evaluated using flowmetry with bilaterally placed laser Doppler probes. Each animal served as its own control, by comparing flow on the manipulated right hemisphere (RH) to that on the unmanipulated LH. Figure 2A shows data for c57BL/6 mice injected with saline (RH), challenged with 6% CO_2_ for 30 seconds and recorded over a 5- minute period. This brief exposure to hypercapnia led to a brisk increase in CBF. The peak increase in CBF was not statistically different in the unmanipulated LH (30.6%) and saline-injected RH (21.3%) of these animals (Figure 2B) (*P* = 0.3; nonparametric permutation test). Figure 2C shows data for mice injected with Tat (RH) and challenged for 30 seconds with 6% CO_2_. The magnitude of the induced change in CBF was significantly lower in the Tat-injected RH (7.3%) versus the non-injected LH (26.3%) (*P* = 0.01; nonparametric permutation test) (Figure 2D). Figure 2E shows data for control mice injected with heat-inactivated Tat (RH) and challenged for 30 seconds with 6% CO_2_. The magnitude of the induced change in CBF was similar in the RH (24.6%) and the unmanipulated LH (29.5%) (Figure 2F). Finally, Figure 2G shows data for mice injected with oligomeric HIV-1 Env (RH) and challenged for 30 seconds with 6% CO_2_. The magnitude of the induced change in CBF was similar in the RH (29.3%) and the unmanipulated LH (32.1%) (Figure 2H).

The data presented in Figure 2 show that, consistent with what was observed in the chronic model, animals acutely exposed to Tat also had a diminished increase in CBF, following exposure to brief hypercapnia. Physiological parameters remained consistent during the 30 second administration of CO_2_ between the WT and Tat-tg mice (Table 1), suggesting that observed increases from baseline CBF (Figure 2) were a result of cerebrovascular responses rather than peripheral hemodynamic accommodations such as increased mean arterial pressure (MAP).

### Cortical capillaries do not show significant changes in length, radius, volume or branching in a model for chronic HIV-associated neuroinflammation

It is generally accepted that cortical blood flow is governed by surface pial arterioles [38], which run along the surface of the cortex, and then dive deeply into the parenchyma of the brain where they form an extensive cortical capillary network. Intracranial delivery of HIV-1 Tat is known to upregulate the release of inflammatory cytokines such as tumor necrosis factor α, interleukin 1β and interleukin-6 within the CNS [39-41]. By doing so, Tat creates a highly inflammatory, and potentially pro-angiogenic, environment (reviewed in [42,43]). This suggests the possibility that chronic HIV-associated neuroinflammation might lead to increased microvascular density and/or increased vessel tortuosity in Tat-exposed mice, which could potentially modulate CVR, and therefore confound the interpretation of our data.

**Figure 3:**
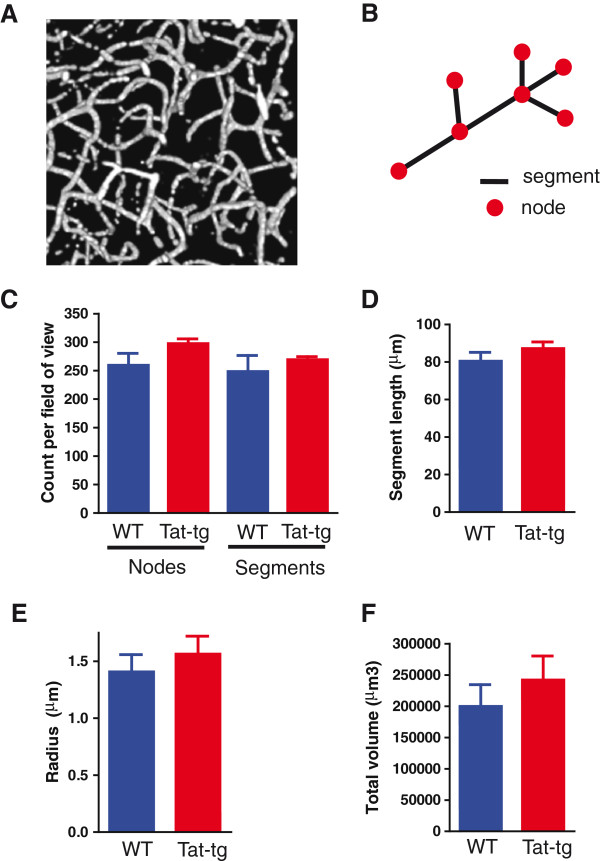
**Cortical capillary morphology is not changed in Tat-transgenic (Tat-tg) mice.** (**A**) Representative image of three-dimensional stacks of cortical tissue capillaries (10x magnification) used for skeletonization with the filament tool in Amira software. (**B**) Schematic of vessel branching used for quantification of morphological features in the skeletonized images. (**C**) The number of nodes and segments in wild type (WT) and Tat-tg mice. (**D**) Mean length of segments. (**E**) Mean radius of vessels. (**F**) Total volume of vessels in one 3-dimensional image stack (500 μm x 500 μm x 100 μm). (C-F) Data extracted from same animals, seven WT and seven Tat-tg. Data represent mean ± SEM. Comparison of cortical capillary parameters from WT and Tat-tg failed to reach statistical significance

To assess whether perturbations of cerebrovascular reactivity might be due to changes in microvessel morphology and density (resulting in impaired flow), we imaged the cortical microvasculature using multiphoton microscopy in Tat-tg, and WT animals. Three- dimensional (500 μm x 500 μm x 100 μm) image stacks were then quantitatively analyzed using Amira software. For each image stack (Figure 3A) a three-dimensional vascular ‘skeleton’ was created by manually tracing vessels in three-dimensional space using the Amira filament tool. Then vessel length, radius, volume and branching parameters (including the numbers of nodes and segments) were extracted. Vessel branching was defined as segments between two nodes where a node is either an endpoint of a vessel or point at which multiple segments arise (Figure 3B). There was no statistically significant difference in the number of nodes and segments between WT and Tat-tg animals (Figure 3C; *P* = 0.120 and *P* = 0.483, respectively; *t*-test). Mean segment length (Figure 3D) was also found to be statistically indistinguishable in the two groups (*P* = 0.279; *t*-test). To determine whether vessel density within the cortex was altered in Tat-tg animals, mean radius (Figure 3E) and total vessel volume (Figure 3F) were calculated. No significant difference between WT and Tat-tg mice was detected (*P* = 0.490, and *P* = 0.431, respectively; *t*-test).

### PDE5 inhibition restores vasodilatory function in the context of chronic HIV-associated neuroinflammation

Hypercapnia is thought to induce vasodilation through the NO-cGMP pathway between perivascular neurons and/or endothelial cells and vascular smooth muscle [19-23]. To test whether this pathway might be dysregulated in the context of HIV-associated neuroinflammation, we conducted hypercapnic challenge experiments in Tat-tg mice that were treated with either 1) tetrahydrobiopterin (BH4), an essential and potentially limiting cofactor in the production of NO [26,27] or 2) an inhibitor of PDE5, which regulates cellular cGMP levels [44]. Physiological parameters were not changed by these treatments (Table 1).

**Figure 4:**
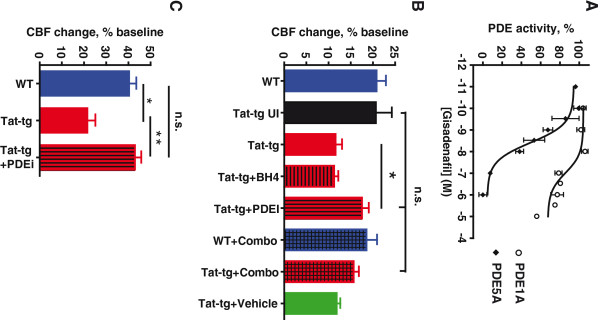
**Phosphodiesterase 5 inhibition restores vasodilatory function in Tat-transgenic mice.** (**A**) Specificity of gisadenafil as demonstrated by measuring phosphodiesterase (PDE) activity in lysates of Cos7 cells overexpressing either PDE1A or PDE5A. (**B**). Maximum cerebral blood flow (CBF) reached in response to 30-second exposure to 6% CO_2_, as measured by laser Doppler flowmetry. The data for the untreated wild type (WT) and Tat-tg and WT mice are taken from Figure 1 (C, D); they are included here for comparison purposes. Number of animals: three for Tat-tg+BH4, five for Tat-tg + PDE5 inhibitor gisadenafil (PDEi), three for WT treated with combination of BH4 and PDEi, three for Tat-tg treated with drug combination, four for Tat-tg injected with vehicle. (**C**). Maximum CBF reached in response to 5-minute exposure to 6% CO_2_. The data for the untreated WT and Tat-tg and WT mice are taken from Figure 1 (A, B); they are included here for comparison purposes. **P <0.01, *P <0.05; n.s., not significant, nonparametric permutation test. All data represent mean ± SEM

Since some PDE5 inhibitors can also interact with PDE1 isotypes found within the cerebral vasculature, we first confirmed the specificity of gisadenafil for PDE5. This was directly tested with recombinant PDE5A and PDE1A overexpressed in COS-7 cells (Figure 4A). Using this approach, we found the IC_50_ of gisadenafil for PDE5A to be 3.6 nM, similar to its reported IC_50_ of 1.23 nM [24]. In contrast, we found the IC_50_ of gisadenafil for PDE1A to be 9.1 μM, an approximately 2500-fold difference in specificity. Thus, gisadenafil at the concentrations used in this study should be specific to PDE5.

Tat-tg animals were treated with BH4 or gisadenafil besylate (UK-369,003; [24,25]) by intraperitoneal injection 2 hours before measurement of the cerebrovascular response to brief hypercapnia. BH4 supplementation did not restore the normal cerebrovascular response to brief hypercapnic challenge (Figure 4B). In contrast, Tat-tg animals treated with the PDE5 inhibitor gisadenafil showed a marked restoration of the normal cerebrovascular response to 30 second hypercapnic challenge (17.5% peak increase in CBF), relative to untreated Tat-tg mice (11.6% peak increase in CBF) (Figure 4B). Combination treatment with both BH4 and gisadenafil had a slightly lesser (15.7%) effect compared to gisadenafil alone (Figure 4B). Longer (5 minute) exposures to CO_2 _ were also performed in animals treated with gisadenafil to determine if these changes to CVR were repro ducible under extended hypercapnia. Tat-tg animals treated with gisadenafil showed marked improvement (*P* = 0.003, nonparametric permutation test) in CVR (42.9% peak increase in CBF) compared to non-treated Tat-tg animals (21.5% peak increase in CBF) (Figure 4C).

### Dilatory capacity of pial arterioles is decreased in a model for chronic HIV-associated neuroinflammation

Since the morphology and gross structure of pial arterioles was unchanged in our model for HIV-associated neuroinflammation, we reasoned that the physical response of pial arterioles to pCO_2_ could be changed due to vessel pathology, and underlie the attenuated cerebrovascular reactivity in Tat-tg mice. We therefore measured changes in the diameter of these vessels in response to brief hypercapnia, by performing in vivo two-photon microscopy in both WT and Tat-tg mice, during CO_2_ administration.

**Figure 5:**
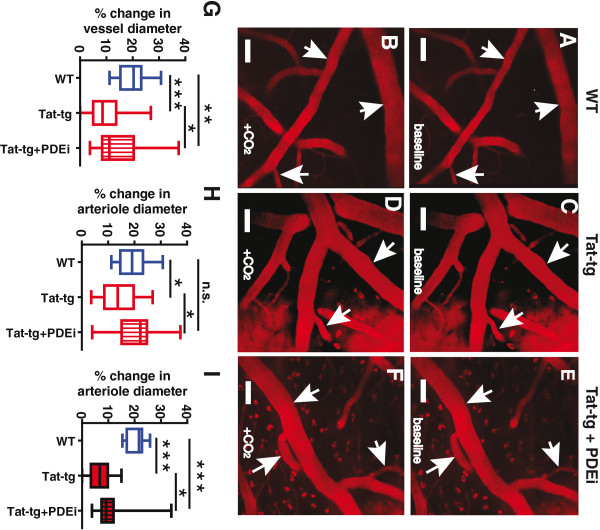
**Dilatory capacity of pial arterioles of Tat-transgenic mice is decreased.** (**A**-**F**) Representative images of dilatory response of pial arterioles in wild type (WT), Tat-transgenic (Tat-tg), and Tat-tg mice treated with PDE5 inhibitor gisadenafil (PDEi), taken before (A, C, E) and after (B, D, F) exposure to 30 seconds of 6% CO_2_. 25X magnification. Arrows point to representative arterioles. Scale bar is 50 micrometers (μm). (**G**) Average magnitude of vessel dilation after exposure to CO_2_ (all vessels; initial diameters 1 to 50 μm). Data shown were collected from seven WT mice (a total of 23 arterioles were analyzed), seven Tat-tg mice (a total of 36 arterioles), and seven Tat-tg mice treated with PDEi (a total of 45 arterioles). (**H**) Average magnitude of vessel dilation after exposure to CO_2_ (small vessels only; initial diameters 1 to 25 μm). These data are a subset of those shown in (G). (**I**). Average magnitude of vessel dilation after exposure to CO_2_ (larger vessels only; initial diameters 26 to 50 μm). These data are a subset of the data shown in (G). **G**-**I**. Data are plotted as box- plots. Maximum and minimum outliers are represented by whisker endpoints. Box segmentation represents lowest datum within 1.5 interquartile range of lower quartile, median and highest datum within 1.5 interquartile range of the upper quartile. Statistical significance denoted as *P <0.05; **P <0.01; ***P <0.001; or n.s., no significant difference; *t*-test

A thinned skull window was prepared over the right somatosensory cortex [34], and Texas Red-dextran was then administered intravenously. Each animal was imaged for 1-minute baseline, then 6% CO_2_ was then delivered for 30 seconds via nose cone and imaging was continued for a total of 5 minutes. Representative images for the peak responses of WT and Tat-tg mice to intermittent hypercapnia are shown in Figure 5. In WT mice, cerebral arterioles, of all sizes dilated as expected in response to brief hypercapnia (Figure 5A,B), whereas in Tat-tg mice, this dilatory response was more attenuated (Figure 5C,D) in larger vessels (>25 μm). Treatment of Tat-tg mice with gisadenafil largely restored the normal vasodilatory response to hypercapnia (Figure 5E,F) by restoring small vessel (<25 μm) dilatory capacity.

These findings were quantitated by measuring the percent change of vessel diameter after exposure to CO_2_, as compared to baseline, using ImageJ software (NIH). Increases in pial vessel diameters (1 to 50 μm) were significantly different (*P* <0.0001; *t*-test) in WT (19.6%) and Tat-tg (10.0%) animals, showing compromise of the normal vasodilatory response to hypercapnia in Tat-tg model (Figure 5G). Interestingly, greater attenuation was observed in the dilation of larger caliber vessels (Figure 5I; >25 μm) (20.5% in WT mice, versus 6.7% in Tat-tg mice; *P* <0.0001; *t-test*) compared to smaller caliber vessels (Figure 5H; <25 μm) (19.1% in WT mice, versus 13.8% in Tat-tg mice; *P* = 0.0251; *t-test*).

We next examined individual arterioles in Tat-tg mice that were treated with gisadenafil. Comparison of all vessels (Figure 5G; 1 to 50 μm diameter) from untreated Tat-tg (10.0% peak increase in vessel diameter) and Tat-tg animals treated with gisadenafil (14.3% peak increase in vessel diameter), showed a significant improvement in dilatory capacity (*P* = 0.0135; *t*-test). However, vessel dilation failed to achieve levels observed for WT mice (19.6% increase in vessel diameter; *P* = 0.0069, *t*-test). Interestingly, smaller arterioles (1 to 25 μm diameter) in Tat-tg mice treated with the PDE5 inhibitor exhibited a robust dilatory response (20.6% increase in vessel diameter) to brief hypercapnia (Figure 5H), which was statistically equivalent (*P* = 0.5447; *t*-test) to the dilatory response of comparably sized vessels in WT animals (19.1% increase in vessel diameter). The dilation of larger arterioles (26 to 50 μm diameter) in Tat-tg mice was also enhanced by treatment with the PDE5 inhibitor (10.6% increase in vessel diameter), compared to untreated Tat-tg mice (6.7% increase in vessel diameter) (*P* = 0.0187; *t*-test). However, the dilatory response of these treated larger vessels was not fully restored when compared to WT mice (20.5%) (Figure 7I; *P* <0.0001; *t*-test).

## Discussion

We used hypercapnia as an experimental tool to examine the regulation of cerebrovascular reactivity in the context of HIV-induced neuroinflammation. Our initial experiments used a relatively extended (5 minute) exposure to moderate hypercapnia, consistent with previous studies in other mouse and rat models of cerebrovascular reactivity (6% CO_2_ stimulus with 9-minute evaluation of CBF) [45-47]. These studies demonstrated a significant loss of normal vascular responsiveness to hypercapnia in Tat-exposed mice, relative to controls (Figure 1). However, we noted that there was considerable acidemia in the animals at the end of this CO_2_ administration period (Table 1). Respiratory acidosis of this kind [18] can stimulate systemic hemodynamic change through β-adrenergic signaling [16,17]. To avoid this, we therefore explored the administration of a 30-second CO_2_ challenge. This shorter hypercapnic exposure recapitulated our initial long exposure findings (compare Figures 1A with 1C), while minimizing the potential confounder of moderate respiratory acidosis (Table 1).

We proceeded to use this hypercapnic challenge model to examine the regulation of CBF in the context of two experimental animal models for HIV-induced neuroinflammation. In both models, mice were exposed to HIV-1 Tat (either by direct intracranial injection or through enforced transgene expression within the CNS), in order to induce a neuroinflammatory state mimicking that found in persons with HIV-associated neurocognitive disorders. In both models, we detected significant reductions in cerebrovascular reactivity to brief hypercapnia, suggesting an underlying cerebrovascular pathology that was induced by exposure to HIV-1 Tat. Mice exposed acutely to intracranial Tat showed a more profound depression in the cerebrovascular response to hypercapnia compared to Tat-tg mice. This difference might be explained by a robust inflammatory response to acutely injected Tat, compared to a more slowly developing, chronic inflammation produced in the Tat-tg mouse during three weeks of DOX-induced Tat expression. Since HIV-associated neuroinflammation is also a chronic disease, all remaining experiments were conducted in the Tat-tg model.

Tat-tg mice showed a 50% attenuation of the normal increase in CBF, following exposure to hypercapnia. This suggests that the dysregulation of CBF in persons living with HIV-1 may be a direct result of virally-induced neurovascular inflammation, and not secondary to peripheral inflammation or peripheral vascular disease associated with HIV-1 infection. Additionally, these findings suggest that decreased resting CBF in persons living with HIV [12] might be explained not only as a result of reduced neuronal demand (due to neuronal damage), but also as the consequence of an HIV-1 induced vascular dysfunction. Clinically, the failure to regulate CBF in the face of changing metabolic demand, such as increased CO_2_ levels, may in turn lead to transient episodes of cerebral ischemia, further perpetuating neuronal damage and inflammation.

In addition to examining regional cerebral blood flow using laser Doppler flowmetry, we also conducted experiments to look at the response of individual vessels to brief hypercapnia. To do this, we conducted in vivo two-photon imaging of cerebral blood vessels, using a thin-skull window (intact cranium). This allowed preservation of normal intracranial pressure, CSF dynamics and hemodynamics. We found that Tat-induced neuroinflammation reduced the overall magnitude of CO_2_-induced vasodilation, with an especially profound effect on larger vessels (>25 μm diameter) (Figure 5).

Cerebrovascular responses to hypercapnia are believed to occur through an NO-cGMP pathway between perivascular neurons and smooth muscle cells [19-23]. This pathway is highly susceptible to dysregulation by reactive oxygen species produced during inflammation [48,49]. Moreover, HIV-1 Tat has also been shown to significantly decrease endothelial NOS (eNOS) expression in porcine coronary arteries [50]. We therefore conducted experiments to examine the role of the NO-cGMP axis in contributing to the diminished vascular response to brief hypercapnia in mice with HIV-1 associated neuroinflammation.

First, we tested whether increasing the supply of NO might correct the vascular response to CO_2_. NO production by NOS is dependent on its cofactor tetrahydrobiopterin (BH4) [26,27]. When BH4 levels are inadequate, the reduction of O_2_ by NOS is no longer coupled to L-arginine oxidation, resulting in generation of superoxide rather than NO. To test whether this uncoupling of NOS was responsible for impaired CVR in our model, we supplemented Tat-tg and WT mice with BH4 and then exposed them to brief hypercapnia. BH4 had no effect on the cerebrovascular responses to brief hypercapnia, which remained attenuated in the Tat-tg mice. In contrast, when Tat-tg mice were treated with a PDE5 inhibitor (gisadenafil), expected to prevent degradation of cGMP in arterial smooth muscle cells [24,25], their cerebrovascular response to brief hypercapnia was normalized. This indicates that CO_2_ chemosensing was not impaired in the Tat-tg mice, and suggests that HIV-associated neuroinflammation may cause cerebrovascular pathology, through effects on cGMP metabolism and possibly PDE5. Interestingly, while gisadenafil produced significant recovery of the normal CBF response to brief hypercapnia in both small and large diameter vessels (Figure 5H, I, respectively), it showed selectivity for smaller vessels (<25 μm). One possible explanation is that the dilatation of the larger vessels is more reliant on NO [51,52], which may not have been adequately replenished with BH4 supplementation. An alternative explanation is that PDE5 expression may be limited to the smaller vessels, whereas other PDE family members exist within the larger arterioles. Future studies will need to address the large vessel responsiveness to different PDE inhibitors. More importantly, however, inhibition of PDE5 was sufficient to restore the normal increase in CVR. This suggests that responses in the smaller vasculature may be of greater importance, compared to larger arterioles (>25 μm), in overall control of CBF following brief hypercapnic stimulation. Additionally, it is possible that already available PDE5 inhibitors could be used to limit further neuroinflammation initiated by transient episodes of tissue ischemia, due to decreased CVR to CO_2_ or other CBF regulators signaling through the NO-cGMP pathway.

Overall, this study reports three major findings. First, HIV-associated neuroinflammation (induced by either acute or chronic exposure to HIV-1 Tat) produced a cerebrovascular pathology, that was uncovered upon brief exposure to moderate hypercapnia. Second, this cerebrovascular pathology may selectively affect larger vessels (>25 μm) and finally, inhibition of PDE5 by gisadenafil besylate restored the normal cerebrovascular response to transient hypercapnia in mice with underlying HIV-associated neuroinflammation by fully restoring the dilatory capacity of the smaller pial arterioles. These findings may have important implications for persons living with HIV-1 especially because impaired vasoreactivity to hypercapnia has been associated with increased risk of ischemic stroke, elevated overall cardiovascular risk and cognitive impairment [53-58].

## Conclusions

This study shows that HIV-associated neuroinflammation can directly result in cerebrovascular pathology. This may explain the dysregulation of cerebral blood flow and cerebrovascular reactivity in persons living with HIV. Our findings also implicate PDE5 as a key mediator of this process, and suggest that pharmacologic inhibition of this enzyme may restore normal cerebrovascular responses. This could have important therapeutic implications for the treatment of HIV-associated neurocognitive disorders.

### Consent

All animal experiments were approved by the University of Rochester Committee on Animal Research, and were conducted in accordance with institutional, federal and local animal use regulations.

## Abbreviations

ABG: arterial blood gases; BH4: tetrahydrobiopterin; CBF: cerebral blood flow; cGMP: cyclic guanosine monophosphate; CNS: central nervous system; CVR: cerebrovascular reactivity; DOX: doxycycline; GFAP: glial fibrillary acidic protein; HAND: HIV-associated neurocognitive disorder; HIV-1: human immunodeficiency virus, type-1; ICI: intracranial injection; IP: intraperitoneal; LDF: laser Doppler flowmetry; LH: left hemisphere; MAP: mean arterial pressure; NO: nitric oxide; NOS: nitric oxide synthase; PDE: phosphodiesterase; RH: right hemisphere; rt-PCR: reverse transcriptase-polymerase chain reaction; Tat-ICI: Tat by direct intracerebral injection; Tat-tg: Tat-transgenic; WT: wild type.

## Competing interests

The authors declare that they have no competing interests.

## Authors’ contributions

JNS carried out in vivo imaging and laser Doppler flowmetry (LDF), helped to conceive the study, and drafted the manuscript. OP conducted in vivo imaging and LDF, and helped to conceive the study. WK conducted experiments to analyze the specificity of gisadenafil for PDE5 versus PDE1. JTZ assisted with surgeries for blood gas and MAP data. MG carried out genotyping and breeding of Tat-tg mice. TL carried out LDF experiments. FO analyzed cGMP levels. CF performed the statistical analysis. CY contributed to the design and conception of experiments to examine the NO/cGMP axis. KAK participated in the design of the study, the performance of in vivo imaging studies, and the analysis of in vivo imaging data. SD conceived of the study, and participated in its design and coordination and helped to draft the manuscript. All authors read and approved the final manuscript.
